# Machine learning-enhanced identification of fluorophilic interactions for improved SERS detection of PFOA

**DOI:** 10.1039/d5en00721f

**Published:** 2026-02-20

**Authors:** Monika Poonia, Kathryn Terceiro, Geoffrey D. Bothun

**Affiliations:** a Department of Civil and Environmental Engineering, Wayne State University Detroit MI 48202 USA monika.poonia@wayne.edu; b Department of Chemical, Biomolecular, and Materials Engineering, University of Rhode Island Kingston RI 02881 USA gbothun@uri.edu

## Abstract

Per- and polyfluoroalkyl substances (PFASs), such as the legacy C8 compound perfluorooctanoic acid (PFOA), pose significant environmental and health risks due to their persistence and widespread use. While surface-enhanced Raman spectroscopy (SERS) has shown promise for PFAS detection, challenges remain in achieving high sensitivity and understanding the underlying molecular interactions. This study combines fluorinated thiol-modified SERS substrates with machine learning techniques to enhance PFOA detection and elucidate fluorophilic interactions. Three different fluorinated thiols were used to modify SERS substrates, and their performance in PFOA detection was compared to bare substrates. Surface modification improved SERS signal enhancement and lowered detection limits compared to unmodified surfaces. Machine learning algorithms, including partial least squares-discriminant analysis (PLS-DA), partial least squares regression (PLSR), and support vector machine (SVM) regression, were employed to classify and quantify PFOA based on Raman spectral features. The PLS-DA model successfully distinguished ligand-specific interaction patterns, demonstrating strong robustness and predictive performance, while SVM regression achieved a limit of detection of 3.61 ppb. We further validated the proposed machine learning-guided SERS approach for PFOA detection directly in water, demonstrating ligand-specific Raman responses arising from fluorophilic ligand–PFOA interactions. A linear and sensitive response was observed at environmentally relevant concentrations, confirming the practical applicability of the approach for aqueous PFAS monitoring. This approach offers a novel SERS-based strategy for detecting PFOA, with results anticipated to inform future developments in applying this strategy to a broader range of PFAS compounds and more complex environmental matrices.

## Abstract 2

Per- and polyfluoroalkyl substances (PFASs), such as the legacy C8 compound perfluorooctanoic acid (PFOA), pose significant environmental and health risks due to their persistence and widespread use.

## Graphical abstract

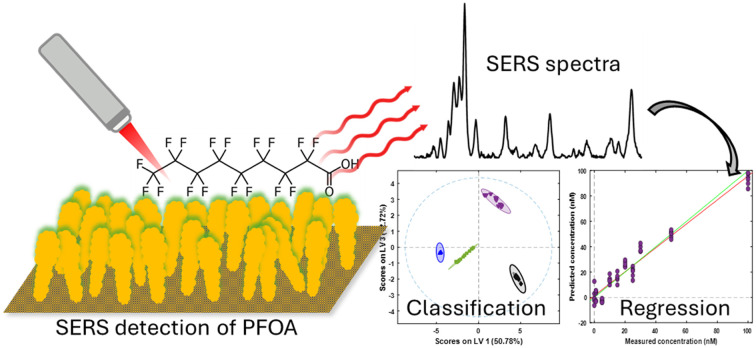

**Environmental significance**

Per- and polyfluoroalkyl substances (PFASs) are a group of persistent, toxic chemicals that pose significant risks to human health and the environment due to their widespread presence in water sources and ecosystems. The detection and quantification of PFAS in environmental samples remain challenging due to their unique physicochemical properties, including high chemical stability and low polarizability. Surface-enhanced Raman spectroscopy (SERS) is a promising technique for the detection of PFAS, but weak interactions between PFAS molecules and traditional SERS substrates lead to poor signal enhancement and limited sensitivity. Aided by machine-learning and using perfluorooctanoic acid (PFOA) as a model PFAS, we have identified fluorophilic interactions between PFAS and functionalized gold nanostructures at nanomolar concentrations that may provide a new approach to selective PFAS detection.

## Introduction

Per- and polyfluoroalkyl substances (PFASs) are a group of persistent, toxic chemicals that pose significant risks to human health and the environment. These substances have been widely used in industrial production and consumer applications, leading to their widespread presence in water sources and ecosystems.^1^ The detection and quantification of PFAS in environmental samples remain challenging due to their unique physicochemical properties, including high chemical stability and low polarizability.^2^ The large number and diversity of PFAS molecules, with varying chemical structures and properties, make it challenging to develop a single detection method that works effectively for all PFAS compounds.^3,4^ Current sensors often lack sufficient sensitivity for PFAS detection at environmentally relevant concentrations.^5^ Improving the interactions between sensor materials and PFAS targets is crucial to addressing these limitations. Among various detection methods, surface-enhanced Raman spectroscopy (SERS) has emerged as a promising technique for the detection of various analytes, including PFAS, due to its high sensitivity and specificity.^6–8^

SERS provides remarkable sensitivity for identifying and analyzing chemical compounds and molecular structures and is widely applicable in the field of environmental monitoring.^9–11^ The Raman signal intensity of analyte molecules can be amplified by a factor of 10^6^ to 10^10^ when they are adsorbed onto specifically engineered plasmonic nanostructures.^12^ However, the detection of PFAS using SERS faces several challenges, including weak interactions between PFAS molecules and traditional SERS substrates, leading to poor signal enhancement and limited sensitivity. Several strategies have been reported to improve sensitivity. Fang *et al.* employed cationic dyes (ethyl violet and methyl blue) to facilitate the adsorption of PFOA from firefighting foams onto graphene oxide mixed with colloidal silver nanoparticles. This method achieved a detection limit of 50 ppb for PFOA.^13^ An improvement in sensitivity was demonstrated using jet-printed silver nanoparticles and graphene on Kapton as SERS substrates with reported PFOA detection at 1 nM or approximately 0.4 ppb.^4^ Detection was attributed to the ability of graphene to adsorb analytes. Park *et al.* developed silver nanograss substrates coated with self-assembled *p*-phenylenediamine nanoparticles, achieving a detection limit of 1.28 pM (0.53 ppt) for PFOA in distilled water.^14^ Feng *et al.* synthesized Ag NP/Au@Ag core–shell nanorod SERS substrates capable of detecting PFOA, PFHxA, and PFBS with a detection limit of 0.1 ppm.^15^ Lastly, Lada *et al.* employed SERS with Ag nanocolloid suspensions to detect methylene blue as an indicator for both short- and long-chain PFAS, achieving a detection limit of 5 ppt.^16^

Recent studies have explored the use of functionalized SERS substrates to improve the detection of PFAS. Rothstein *et al.* enhanced silver nanorod (AgNR) substrates through alkanethiol functionalization for SERS applications, achieving limit of detections (LODs) of 1 ppt for PFOA and 4.28 ppt for PFOS.^7^ The incorporation of fluorinated compounds into SERS substrates has shown potential in enhancing the capture of PFAS through fluorous interactions.^17^ Fluorophilic interactions can potentially enhance the affinity between PFAS molecules and SERS substrates, leading to improved detection sensitivity. Fluorinated thiols have shown promise in leveraging both fluorophilic and electrostatic interactions for PFAS capture.^18–20^ However, the underlying mechanisms of these interactions and their impact on improving SERS detection remain poorly understood. The complexity of PFAS–substrate interactions and the vast number of possible fluorinated thiol structures make it challenging to identify optimal combinations using traditional experimental approaches alone. Machine learning (ML) algorithms have demonstrated their ability to handle complex datasets, consider variable interactions, and build predictive models in various scientific domains.^6,21,22^ The integration of ML with SERS offers the potential to extract meaningful information from complex spectral data and provide insights into the underlying molecular interactions.^6,7,23^ For example, Rothstein *et al.* showed that combining Raman and SERS spectroscopies with ML, specifically support vector machine and support vector regression models, enabled classification of PFOA, PFOS, and reference samples with up to 95% accuracy and achieved low detection limits for both PFOA and PFOS.^7^ However, there is a gap in applying supervised ML methods specifically to optimize fluorophilic interactions for SERS-based PFAS detection.

In this study, we present an innovative strategy that integrates fluorinated thiol-functionalized SERS substrates with ML techniques to improve the detection and analysis of fluorophilic interactions. The fluorinated species exhibit overlapping vibrational bands, and small interaction-driven spectral shifts cannot be reliably isolated using conventional SERS spectral inspection. ML-based classification and regression therefore provide a quantitative means to distinguish subtle yet chemically meaningful variations arising from fluorophilic interactions.^24^ The goal is to develop a foundational SERS-based method for detecting PFAS in environmental samples, initially focusing on PFOA as a representative target compound. We systematically explore various fluorinated thiols as surface modifiers and evaluate their effectiveness in detecting PFOA compared to unmodified SERS substrates. A commercially available gold-coated nanostructured SERS platform, selected for its reproducibility and robustness, serves as the sensing surface.^25,26^ By leveraging ML algorithms such as partial least squares-discriminant analysis (PLS-DA), partial least squares regression (PLSR), and support vector machine (SVM) regression, we aim to elucidate the interaction mechanisms between the fluorinated thiols and PFOA. We assess the analytical capability, sensitivity, and detection limits of the proposed method, which is essential before applying the technique to more complex sample types. Ultimately, this work lays the groundwork for expanding the methodology to a broader spectrum of PFAS compounds and transitioning toward the analysis of complex, real-world environmental samples in future investigations.

## Experimental

### Materials

Perfluorooctanoic acid (C_8_HF_15_O_2_) was obtained from Accustandard, Inc (New Haven, CT). 4-Trifluoromethylbenzyl mercaptan (C_8_H_7_F_3_S) (**TFMB**), 4-trifluoromethyl-2,3,4,5,6-tetrafluorothiophenol (C_7_HF_7_S) (**TFMtFTP**) and 3,3,4,4,5,5,6,6,7,7,8,8,8-tridecafluoro-1-octanethiol (CF_3_(CF_2_)_5_CH_2_CH_2_SH) (**TDFOT**) were purchased from Sigma-Aldrich. The fluorination degree of TFMB, TFMtFTP, and TDFOT are 3, 7 and 13, respectively. Methanol (CH_3_OH, >99.5%) and ethanol (C_2_H_6_O, >99.5%) from Fisher Scientific (Waltham, MA) were used as solvents for making stock solutions of PFOA and thiols. All materials were used as received. Ultrapure MilliQ water (resistivity >18.2 MΩ cm at 25 °C) was obtained from a Millipore Direct-Q3 UV purification system (Billerica, MA).

### SERS substrates and surface modification

SERS substrates with gold-coated silicon nanopillars with a dimension of 3 × 3 mm^2^ were purchased from Silmeco ApS (Copenhagen, Denmark). As reported earlier,^25,26^ the substrate surfaces consisted of highly uniform gold-coated silicon pillars having an effective diameter of about 100 nm with a pillar density of about 18–20 pillars per μm^2^. Nanopillars were leaned together by adding a droplet of deionized water to the substrate and drying the substrates under ambient conditions for 30 min. Substrates were treated with an oxygen-plasma cleaner (Diener electronic, Germany) for 5 min at 30 W and 0.2 Torr to remove organic contaminants and impurities from the surface and activate the adsorption sites through bombardment with energetic ions. Plasma treatment effectively minimizes structural background in the SERS spectra and ensures substrate reproducibility and reliability, which is critical for low-level SERS detection.^27^ Electron microscopy images of SERS substrates were obtained with a Zeiss Sigma VP field emission scanning electron microscope (FE-SEM) using an Everhart–Thornley secondary electron detector at an accelerating voltage of 7 kV.

Surface modification of the substrates was carried out by forming self-assembled monolayers (SAMs) on plasma cleaned and pre-leaned SERS substrates. Three distinct thiol solutions were prepared in ethanol, each at a concentration of 1 mM. The cleaned substrates were immersed in 10 mL of the respective thiol solutions for a duration of 24 h. A close-packed thiol monolayer is expected to have between 4–6 molecules per nm^2^.^28,29^ Using 5 molecules per nm^2^ as a conservative estimate gives a required number of 4.5 × 10^13^ molecules to form a monolayer on the substrate. The 1 mM solution in 10 mL contains approximately 6 × 10^18^ molecules or 1.3 × 10^5^ times the number needed for monolayer coverage. Thus, the chosen 1 mM concentration provides a large molar excess and was selected to favor rapid assembly and high surface coverage. After the incubation period, the substrates were thoroughly rinsed with ethanol to remove any unbound thiol molecules from the surface and dried under ambient conditions for 30 min. We used bare gold-coated substrates as controls for comparison with the functionalized substrates.

### XPS analysis

Surface elemental composition was analyzed by X-ray photoelectron spectroscopy (XPS). The K-Alpha system from Thermo Fisher Scientific equipped with a monochromatic Al Kα source (photon energy: 1486.7 eV, spot size: 400 μm) was used. The investigation of elemental composition was conducted using the survey scan function. For the XPS analysis, spectra were used without any spectral modification.

### Raman instrumentation and spectral measurements

SERS measurements were conducted using a SIERRA 2.0 Raman spectrometer (Snowy Range Instruments, Wyoming) equipped with a 785 nm laser. Spectra were collected in the wavenumber range 200–2000 cm^−1^ with a spectral resolution of 4 cm^−1^. Measurements were conducted with a 100 mW laser power with a spot diameter of ∼40 μm over an integration time of 10 s. Orbital raster scanning (ORS) was used for all measurements providing an effective SERS detection area of 2 mm in diameter. PFOA samples were prepared in methanol at concentrations ranging from 0 to 1 μM. To assess reproducibility, we performed five to six consecutive measurements at the same PFOA concentration and measured standard deviation on the SERS intensity to represent measurement variability.

### PFOA detection in water

A series of PFOA solutions were prepared in deionized water at concentrations between 0 and 1 μM. TDFOT-functionalized substrates were placed inside a PTFE flow device, and 2 mL of each sample was introduced into the flow channel.^30^ For each concentration, five consecutive measurements were taken after adding the solution to the device. The samples were analyzed in order of increasing PFOA concentration, with water rinsing performed between each run.

### Spectral processing and analysis

All spectra were baseline corrected using a rolling circle filter (RCF) background subtraction code^31^ with a radius of 100 000 and further normalized to the maximum intensity. Background-subtracted spectra were analyzed with OriginPro (Version 2019b, OriginLab Corp., Northampton, MA) and MATLAB (MATLAB R2022a, The MathWorks, Inc.). Partial least squares regression discriminant analysis (PLS-DA) model was constructed using 10-fold Venetian blind (VB) cross-validation method in MATLAB with PLS_Toolbox 8.7.1 (Eigenvector Research, Inc. Manson, WA, USA). PLS2-DA was applied to construct a single multi-class model.^32^ The optimal number of latent variables (LVs) for each model was used as per the model suggestions, based on appropriate root mean square error (RMSE) value for cross-validation. To quantitatively assess PFOA concentrations across different substrate treatment conditions and measure LODs, linear regression models were developed using the VB cross-validation method. For comparative analysis, support vector machine (SVM) regression models were also implemented, utilizing the same VB cross-validation approach to ensure methodological consistency.

## Results and discussion

### XPS analysis of thiol modified substrates

**Fig. 1:**
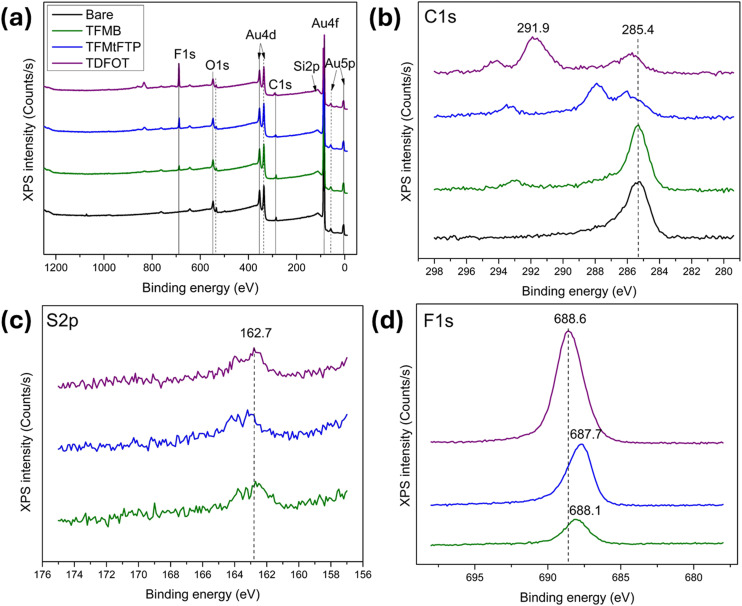
Full-scan XPS survey spectrum (a) and high-resolution XPS spectra of (b) C 1s, (c) S 2p, and (d) F 1s for the bare gold-coated substrate (black) and SERS substrates functionalized with thiols: TFMB (green), TFMtFTP (blue), and TDFOT (purple).

XPS was employed to characterize the SERS substrates following thiol functionalization. Fig. 1a shows the full XPS survey spectra for all four SERS substrates including bare gold-coated substrate (black) and substrates functionalized with TFMB (green), TFMtFTP (blue), and TDFOT (purple). The survey spectra exhibit characteristic peaks at binding energies of 84.8–88.4 eV, 162.7 eV, 285–291.2 eV, 531 eV, and 688.6 eV, corresponding to Au, S, C, O, and F, respectively, confirming successful surface modification.^33,34^ The atomic compositions of Au 4f, O 1s, C 1s, F 1s, and S 2p on the functionalized substrates, as quantified by XPS, are summarized in Table 1.

**Table 1:** Atomic percentage of elements detected by XPS analysis for bare gold-coated and thiolated SERS substrates

	Atomic%			
Element	Bare	TFMB	TFMtFTP	TDFOT
Au 4f	43.48	39.29	34.47	22.44
O 1s	15.68	9.28	7.44	5.62
C 1s	36.68	35.14	25.25	24.75
F 1s	—	14.76	29.55	45.42
S 2p	—	1.32	3.28	1.77

Fig. 1b–d show the high-resolution XPS spectra of C 1s, S 2p, and F 1s, respectively. The C 1s spectra (Fig. 1b) exhibit a dominant peak at ∼285.4 eV for both the unmodified and TFMB-modified substrates, corresponding to carbon. In contrast, the C 1s peaks for the TFMtFTP and TDFOT modified substrates show a slight shift toward higher binding energies, indicating changes in the chemical environment of carbon upon thiol functionalization. Additional features in the 290–292 eV range are attributed to oxidized carbon species, such as carbonyl (C=O) or carboxyl (O–C=O) functional groups, consistent with reported C 1s assignments in this energy region.^35–37^

Sulfur was detected in all thiol-modified substrates through the presence of an S 2p peak at ∼162.7 eV (Fig. 1c), a characteristic signature of thiolate bonding to gold.^38^ This sulfur signal is absent in the unmodified substrate, further confirming successful surface modification. Atomic percentage analysis also detected S 2p exclusively in the functionalized substrates, providing strong evidence for the formation of self-assembled monolayers on the gold surface. The F 1s spectra (Fig. 1d) reveal a fluorine peak in the 687.7–688.6 eV range for all thiol-modified substrates,^39–41^ while no fluorine signal is observed for the unmodified SERS substrate. This peak arises from the fluorinated moieties present in the thiol ligands, confirming their attachment to the substrate surface. A slight shift in F 1s binding energy toward lower values is observed for the TFMB- and TFMtFTP-modified substrates. In addition, the TDFOT-modified substrate exhibits a notably higher fluorine atomic percentage (Table 1), consistent with its fluorine-rich molecular structure.

### SERS spectra of selected thiol compounds

**Fig. 2:**
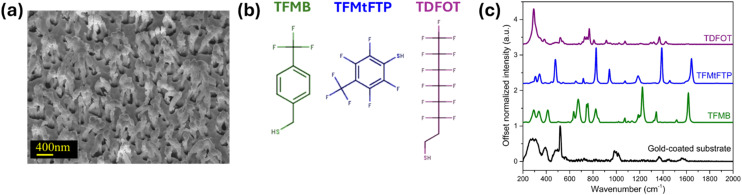
(a) SEM image of pre-leaned nanopillars on SERS substrate. (b) Structure of thiol compounds selected for the substrate surface modification. (c) SERS spectra of gold-coated SERS substrate (black) and SAMs of thiol compounds on to SERS substrates.

Gold-coated freestanding nanopillars serve as effective SERS substrates by concentrating analyte molecules in close proximity to areas of intense electromagnetic field enhancement, known as hot spots.^11^ An SEM image of a SERS substrate after leaning the nanopillars is shown in Fig. 2a. Fig. 2b shows the chemical structures of selected thiol compounds for surface modification. Among these, TFMB is characterized by a benzene ring featuring both a trifluoromethyl (–CF_3_) group and a thiol (–SH) group. TFMtFTP shares structural similarities with TFMB, comprising an aromatic ring with fluorine atoms and a trifluoromethyl group. In contrast, TDFOT's structure diverges from the aromatic pattern, instead consisting of a fluorinated alkyl chain terminated by a thiol group.

The SERS spectra of a bare gold substrate and substrates modified with thiol SAMs are shown in Fig. 2c. The SERS spectrum of gold-coated silicon substrate shows a sharp silicon peak at 520 cm^−1^ along with a broad, low-intensity background due to the gold surface.^25^ The three thiol compounds exhibited robust Raman signals, indicating successful chemisorption on the gold-coated SERS substrate due to the strong affinity between the thiol groups and the gold surface, resulting in the formation of stable gold–sulfur (Au–S) bonds. Each thiol compound has a unique spectral signature in the fingerprint region (200–1800 cm^−1^), reflecting their unique molecular structures. The detailed peak assignments, corresponding vibrational modes, and relevant literature references are summarized in Table S1 in the SI. In the lower wavenumber region, prominent bands observed at 311, and 337 cm^−1^ correspond to Au–S bending modes, indicating strong chemisorption of the sulfur groups on the gold surface.^42^ The bands near 412 and 477 cm^−1^ arises from C–S stretching vibrations, further supporting the formation of a stable thiolate–gold interface on the functionalized substrates.^42,43^ The spectral peaks observed at 290 and 382 cm^−1^ are attributed to C–C and CF_2_ vibrational modes, respectively.^24,44^

Key features in TFMB SERS spectrum include strong peaks around 1000–1600 cm^−1^ due to aromatic ring vibrations.^45^ The peak near 1000 cm^−1^ is indicative of the benzene ring breathing mode and the peak at 1616 cm^−1^ is attributed to C=C stretching vibrations within the benzene ring. A range of signals is observed between 1100–1350 cm^−1^, corresponding to C–F stretching vibrations and a notable strong peak at 1222 cm^−1^ is associated with the C–F stretching vibration specific to the trifluoromethyl group.^45,46^ Potential C–S stretching vibrations appeared in the 600–800 cm^−1^ region.^43,45,46^

The SERS spectrum of TFMtFTP exhibited notable similarities to that of TFMB, with the dominant spectral characteristics primarily originating from the aromatic ring and trifluoromethyl moieties. Subtle variations in peak positions were observed, likely due to the specific location of the trifluoromethyl group on the molecule. TFMtFTP exhibited pronounced features at 828 cm^−1^ and 1390 cm^−1^ corresponding to the symmetric C–F stretching modes of its tetrafluorinated benzene ring, and at 1643 cm^−1^ attributed to C–C stretching in benzene ring. The increased fluorination in TFMtFTP resulted in a more pronounced SERS signal intensity compared to TFMB, highlighting the influence of the electronegative fluorine atoms on the overall SERS response.^47–49^

The SERS spectrum of TDFOT modified surfaces exhibited characteristic peaks at 1360 cm^−1^ and 1420 cm^−1^, attributed to C–F stretching vibrations in the fluorinated alkyl chain. The thiol group C–S stretching mode is observed in the 640–700 cm^−1^ range.^43^ The spectral features corresponding to the C–C and CF_3_ stretching vibration is observed in the 720–767 cm^−1^ region.^7^ Despite its extended perfluorinated chain, TDFOT likely contributes minimally to the overall SERS signal intensity due to its weaker Raman scattering compared to the aromatic structures found in TFMB and TFMtFTP. Aromatic thiols on gold surfaces exhibit sharp and intense spectral peaks, particularly associated with aromatic ring vibrations and C–S stretching, which reflect strong π-system interactions and significant charge transfer at the metal–ligand interface. In contrast, long-chain fluorinated thiols display broader and less intense peaks dominated by C–F stretching and backbone modes, as a result of their distinct packing arrangements and comparatively weaker interactions with the gold substrate.^47–51^ Additionally, it is important to note that the substrate restructuring upon thiol adsorption-especially for long-chain thiols has been reported, leading to changes in the gold surface morphology and electronic properties.^50,52^

### SERS measurement of PFOA on thiolated substrates

**Fig. 3:**
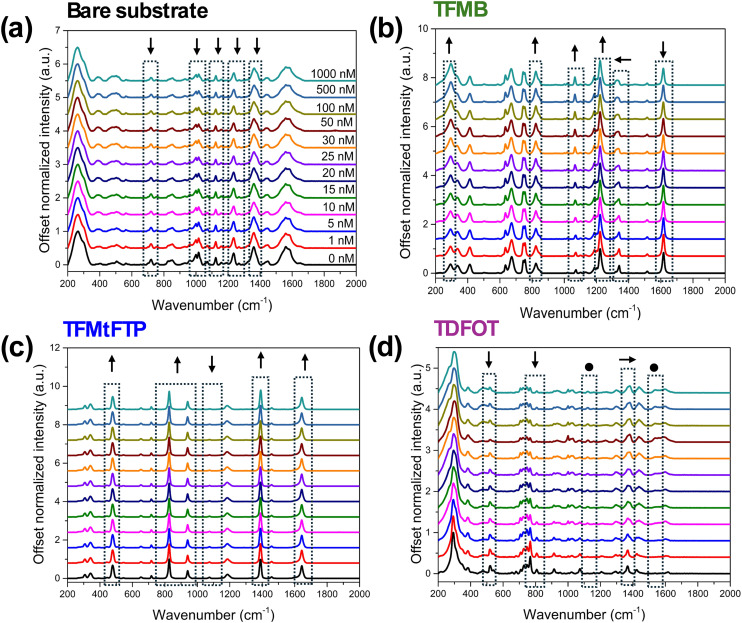
The SERS spectra of PFOA in methanol at the concentrations (from bottom to top) of 0 (reference), 1, 5, 10, 15, 20, 25, 30, 50, 100, 500, and 1000 nM, respectively, on to (a) gold-coated substrate, (b) TFMB (c) TFMtFTP and (d) TDFOT thiol-modified substrates. Inset: Arrows denote spectral changes induced by PFOA: (↑) intensity increment, (↓) intensity decrement, (←) red shift, (→) blue shift, and (•) emergence of a new peak.

A methanol solution of PFOA was prepared at various concentrations ranging from 0 to 1000 nM and a 5 μL droplet of PFOA solution with increasing concentrations was deposited onto the non-modified and thiol-modified substrates. Methanol was chosen primarily due to the enhanced solubility and stability of PFOA in this solvent, which facilitates accurate and reliable quantification by minimizing matrix interferences that are often encountered in more complex samples. The PFOA sample droplets were dried on to modified substrates under ambient conditions for 20 minutes and after which multiple SERS spectra were collected for each PFOA concentration ranging from 0 to 1 μM. In Fig. 3, the average SERS spectra of measured PFOA concentrations on gold coated and three different SAM of thiol substrates are shown.

The SERS analysis of PFOA on gold-coated substrates (Fig. 2a) showed interesting spectral changes as the concentration of PFOA increased. At 0 nM PFOA concentration, several peaks were observed in the SERS spectrum. These peaks can be attributed to gold background and methanol signals. The presence of these background peaks is consistent with previous studies that have reported Raman signals from gold nanostructures and methanol on SERS substrates.^25,53,54^ The intensity of the peaks attributed to gold and methanol initially showed an increment followed by a gradual decrement as the PFOA concentration increased (Fig. S1a in SI). PFOA molecules may adsorb onto the gold surface, altering the plasmonic properties and consequently affecting the background signal intensity.^14^ The presence of PFOA might also influence the orientation or interaction of methanol molecules with the gold surface, leading to changes in the methanol SERS signal.^4^

PFOA itself has relatively weak Raman scattering, making direct detection challenging.^13^ The fluorinated thiols create a functional layer on the SERS substrate that increases the affinity for PFOA through hydrophobic and fluorine–fluorine interactions.^16^ As discussed in Fig. 2c, the thiol molecules themselves act as SERS reporters, providing strong and distinct spectral features. While these signals are stronger than those of PFOA, they serve as a sensitive backdrop against which small spectral changes induced by PFOA can be detected.^4^ By using the thiols as intermediary sensors, we leverage their strong SERS activity to indirectly probe PFOA. The presence of PFOA alters the local environment of the thiol molecules, leading to subtle changes in their SERS spectra. These changes, such as peak shifts, intensity variations, or the appearance of new features, indirectly indicate the presence and concentration of PFOA.^7^

The SERS spectra revealed notable changes upon the addition of PFOA to the functionalized substrates. For the TFMB-modified surface, as illustrated in Fig. 3b and S1b, a gradual increase in intensity was observed for peaks associated with C–F stretching (1074 and 1222 cm^−1^) vibrations as PFOA concentration increased. Conversely, the intensity of the C=C stretching band at 1616 cm^−1^ decreased with increasing PFOA concentration. Interestingly, another C–F stretching peak exhibited a decrease in intensity and a slight shift from 1340 cm^−1^ to 1329 cm^−1^. This shift suggests a potential interaction between the fluorinated groups of TFMB and PFOA. Similarly, the TFMtFTP-functionalized substrate displayed comparable sensitivity to PFOA. As shown in Fig. 3c and S1c, the intensities of peaks corresponding to C=C stretching and C–F stretching vibrational modes also increased gradually with rising PFOA concentration. Interestingly, another C–F stretching peak at 1074 cm^−1^ exhibited a decrease in intensity.

The TDFOT-modified substrate exhibited a distinct response to PFOA compared to the other functionalized surfaces. Notably, a significant decrease in peak intensities was observed in the lower wavenumber region (400–1100 cm^−1^). In the 650–800 cm^−1^ region, the Raman features of TDFOT do not decrease uniformly with increasing PFOA concentration. Instead, mode-specific behavior is observed. The peak at ∼767 cm^−1^, assigned to the CF_3_ stretching vibration, shows the largest and most consistent decrease, indicating strong perturbation by PFOA adsorption. In contrast, the neighboring bands at 720–748 cm^−1^, associated with C–C and CF_3_ stretching modes, exhibit smaller or non-monotonic changes, the 720 cm^−1^ band decreases only slightly, while the 748 cm^−1^ mode initially increases before decreasing at higher concentrations. These inconsistent variations reflect differences in how each vibrational mode interacts with the local chemical environment.^55^ In the higher wavenumber range (1150–1616 cm^−1^), the peaks became broader and showed either red or blue shifts of approximately 10 wavenumbers. These spectral changes suggest the occurrence of hydrophobic interactions between the fluorinated chains of TDFOT and PFOA. The TDFOT thiol, which shares structural similarities with PFOA, also demonstrated new peaks emerging around 1120 and 1538 cm^−1^ in the presence of PFOA (Fig. 3d). The intensity of this peak increased with the PFOA concentration, indicating a direct correlation between the spectral response and the amount of PFOA present. These observations highlight the sensitivity of the TDFOT-functionalized substrate to PFOA and suggest that the structural similarity between TDFOT and PFOA may contribute to enhanced detection capabilities.^56^ Small error bars for the main Raman peak intensities, shown in Fig. S1a–d, demonstrate the high reproducibility and consistency among SERS spectra collected at the same PFOA concentration.

The high electronegativity and low polarizability of fluorine atoms create an electron-deficient environment along PFOA's fluorocarbon (C–F) chain.^57^ This environment drives selective interactions with electron-rich thiol groups, through a synergistic interplay of dipole–dipole forces and lone pair–π interactions, collectively termed as C–F⋯F–C intermolecular dispersion interactions, or fluorophilic interactions.^20,57,58^ These fluorophilic interactions enable PFOA to preferentially adsorb onto thiol-functionalized surfaces, thereby concentrating the analyte near the SERS substrate's plasmonic hotspots.^58^

### Partial least squares-discriminant analysis

To further investigate the spectral differences and interaction between thiols and PFOA, partial least squares-discriminant analysis (PLS-DA) was applied. PLS-DA considers the entire spectral profile rather than individual peaks, capturing subtle spectral changes induced by PFOA interactions. The mean-centered, baseline-corrected, normalized SERS spectra within the 200–2000 cm^−1^ fingerprint wavenumber range from the four groups are used as the descriptor (*X*) matrix, whereas the response (*Y*) vector was created to indicate group identities. PLS-DA optimizes the fit between the descriptor matrix and class groups by maximizing covariance, resulting in the determination of latent variables (LVs) in terms of the PLS score.^59,60^

**Fig. 4:**
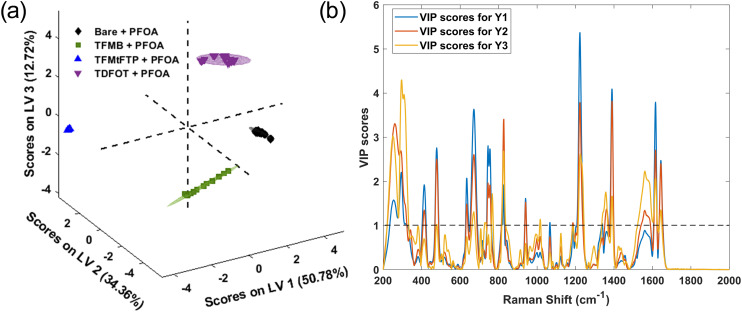
(a) PLS-DA score plots for PFOA spectral data with varying concentrations on bare and three different thiol-modified substrates. (b) Variable importance in projection (VIP) score plots highlighting important discriminative wavenumber bands between control and thiolated groups. Black dotted line represents the significance threshold.

The classification models incorporated three latent variables: LV1, LV2, and LV3, which accounted for 50.78%, 34.36%, and 12.72% of the variance, respectively. As illustrated in Fig. 4a, these three LVs effectively differentiated all four types of surface treatments and captured the majority of the variance. The PLS-DA model's performance was evaluated using specificity, sensitivity, and root mean square error (RMSE) metrics, as presented in Table 2. The results demonstrated the model's high effectiveness and robustness, achieving clear classification of sample groups (with sensitivity and selectivity values of 1).

**Table 2:** Partial least squares-discriminant analysis (PLS-DA) model results on bare gold-coated and thiolated SERS substrates

Surface treatment	Specificity	Sensitivity	RMSE
Bare (*n* = 69)	1.00	1.00	0.058
TFMB (*n* = 71)	1.00	1.00	0.023
TFMtFTP (*n* = 72)	1.00	1.00	0.049
TDFOT (*n* = 69)	1.00	1.00	0.056

The variable importance in projection (VIP) scores were used to measure the influence of the initial *X* variables (*i.e.*, Raman shift) on the classification model. A VIP score in PLS-DA is a metric that estimates how important a variable is in a PLS-DA model. A variable with a VIP score close to or greater than 1 indicates that the spectral bands and wavenumbers are discriminative between the two or more groups. Fig. 4b shows the VIP score evaluated from the PLS-DA classification model. As evident from the spectral difference between all four surface treatments and PFOA interactions, the VIP score also shows that the aromatic and aliphatic types of thiols and PFOA conformations are the most discriminative in the model. From the VIP score that is evaluated using the PLS-DA classification model, the spectral regions at 415 cm^−1^, 477 cm^−1^, 633–671 cm^−1^, 745 cm^−1^, 826 cm^−1^, 1222 cm^−1^, 1360–1390 cm^−1^, 1560 cm^−1^ and 1616–1644 cm^−1^ exhibit VIP scores greater than one. These bands are discussed in detail in the previous section and consistent with the vibrational assignments reported by Cho *et al.*^24^ and Kumar *et al.*,^61^ whose experimental and DFT-supported analyses provide reference positions for C–F stretching, CF_2_ deformation, and CF_3_ modes. This demonstrates that these wavenumbers serve as discriminative spectral features for distinguishing the thiol groups in the PLS-DA classification model.

The receiver operating characteristic (ROC) curve was also plotted to examine the efficiency and performance of the PLS-DA classification model. The ROC curve was constructed between 1-specificity (false positive rate) and sensitivity (true positive rate) for each surface modification, and the efficiency of the PLS-DA model is illustrated by the area under the curve (AUC). Fig. S2 in SI shows the ROC with an AUC value of 1.00, showing the fitting model with high accuracy as an AUC near to zero indicates an inaccurate model. PLS-DA showed that the SERS data sets for the different surface treatment and PFOA interactions are well differentiated and provided a best fit model to discriminate the interaction process of PFOA. While the presented SERS-based detection method demonstrates promising performance under controlled conditions, it is important to acknowledge that environmental samples often contain potential interferents such as chloride, sulfate, and surfactants, which may affect signal intensity and reproducibility. Although the functionalized thiol layer was designed to enhance analyte–specific interactions and minimize nonspecific binding, the presence of structurally similar fluorinated compounds (*e.g.*, PFOS) may still lead to spectral overlap and reduced classification specificity. This limitation highlights the need for future studies to incorporate a broader range of co-existing substances to assess robustness and selectivity in more complex matrices.

To assess whether chemometric classification can reliably differentiate PFOA-exposed substrates from unexposed substrates, independent of surface ligand chemistry, we performed PLS-DA separately on all four substrate conditions using spectra collected without and with PFOA in methanol (Fig. S4). Across all substrates, the PLS-DA score plots show a clear two-class separation with well-defined clustering of the methanol and PFOA samples. The corresponding cross-validated *Y*-prediction plots further confirm the robustness of this discrimination, showing very little to no overlap between classes for each substrate type (Fig. S5). These results demonstrate that the model identifies PFOA-specific spectral features that are consistent across all ligand chemistries and are not driven by inherent differences between the substrates themselves.

### Partial least square regression analysis

Partial least squares regression (PLSR) was employed for quantitative analysis to predict PFOA concentrations across various surface modifications. This regression technique was used to model the relationship between the SERS spectra (*X*) and the corresponding PFOA concentrations (*Y*). To assess the model's generalization capability, venetian blinds (VB) cross-validation was implemented. The 10-fold VB cross-validation ensures that the model performs well on unseen data and is not overfitting to the training set. Additionally, VIP scores were utilized to examine the spectral features most relevant to the regression. VIP scores provide valuable insights into the chemical changes associated with PFOA interactions by highlighting the most influential spectral regions.

**Fig. 5:**
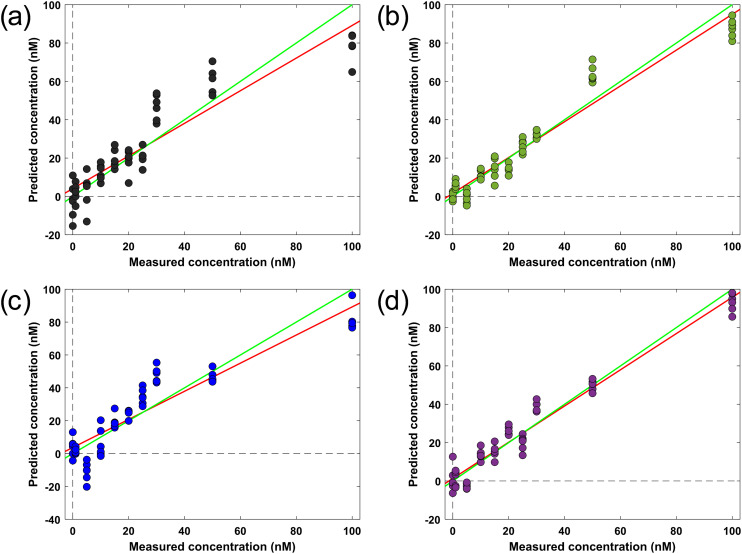
Cross-validation parity plots showing the predicted concentration *versus* the true concentration using partial least-squares (PLS) regression in PFOA spectra on (a) bare substrate (b) TFMB (c) TFMtFTP and (d) TDFOT modified substrates. The fit line (red) and 1 : 1 line (green) represents the overall trend of the model's predictions. Each dot is a graphical representation of an individual sample projected in latent variable space.

The validity of the PLSR model was evaluated in terms of method linearity and accuracy. The linearity of the method was represented in terms of the correlation coefficient (*R*^2^) between the actual concentration of PFOA and the predicted values from the successive PLSR model for the test samples. The PLS regression models demonstrate varying degrees of effectiveness in PFOA detection across the different surface modifications (Fig. 5). The bare gold-coated substrate showed moderate performance with an *R*^2^ of 0.83 and root-mean square error cross validation (RMSECV) of 11.09 (Fig. 5a). This suggests that while the unmodified surface can detect PFOA, there is room for improvement in terms of prediction accuracy and precision. Among the thiol-modified surfaces, TDFOT demonstrated the best performance with the highest *R*^2^ (0.95) and lowest RMSECV (6.09) (Fig. 5d). The RMSECV is a measure of the model's predictive performance and a lower RMSECV indicates a more accurate and precise predictive model. This indicates that TDFOT modification significantly enhances the substrate's ability to detect and quantify PFOA concentrations with high accuracy and precision. TFMB modification also showed excellent results (Fig. 5b), with an *R*^2^ of 0.93 and RMSECV of 7.44, suggesting that it too provides a substantial improvement over the bare substrate for PFOA detection. As illustrated in Fig. 5c, TFMtFTP modification showed the least detection capabilities among the modified surfaces, with an *R*^2^ of 0.84 and RMSECV of 11.45.

The support vector machines (SVM) regression models (SVR) depicted in Fig. S3 exhibit distinct performance levels in PFOA detection across various surface modifications. Analysis showed RMSECV values of 8.06 (bare substrate), 7.77 (TFMB), 5.67 (TFMtFTP), and 2.88 (TDFOT-modified substrate), indicating progressively improved accuracy. Table 3 compares the detection limits (parts per billion or μg L^−1^; 1 ppb = 2.42 nM PFOA) derived from PLS and SVM regression models for each modification. The detection limit for both models was estimated using the validation dataset and defined as the lowest concentration for which the model's prediction error is smaller than the analyte concentration and statistically distinguishable from the blank within the 95% confidence interval of the calibration residuals. This approach is consistent with commonly used chemometric criteria for multivariate calibration models.^62,63^ PLS regression yielded detection limits of 13.77 (bare), 9.248 (TFMB), 14.22 (TFMtFTP), and 7.56 ppb (TDFOT), whereas SVM regression achieved enhanced sensitivity with limits of 10.02, 6.44, 7.08, and 3.60 ppb, respectively. These results highlight SVR's superior performance in lowering detection thresholds across all substrate modifications. At low PFOA concentrations, a small number of PLSR-generated predictions fell below the blank, which is attributed to model-dependent sensitivity to spectral noise and baseline fluctuations. PLSR, particularly when multiple latent variables are retained, can partially overfit low-signal regions, producing slight negative deviations.^64^ In contrast, SVM regression applied to the same dataset did not exhibit this behavior (Fig. S3) and yielded superior sensitivity limits, reflecting its stronger regularization and reduced susceptibility to noise. A detailed comparison table (Table S2 in the SI) presents our LOD results alongside those reported for similar SERS-based techniques and liquid chromatography-mass spectrometry (LC-MS) methods in recent studies.

**Table 3:** A comparison of detection limits (parts per billion, ppb) derived from PLSR and SVR models on bare gold-coated and thiolated SERS substrates

	Limit of detection (ppb)			
	Bare	TFMB	TFMtFTP	TDFOT
PLSR	13.77	9.24	14.22	7.56
SVR	10.02	6.44	7.08	3.60

**Fig. 6:**
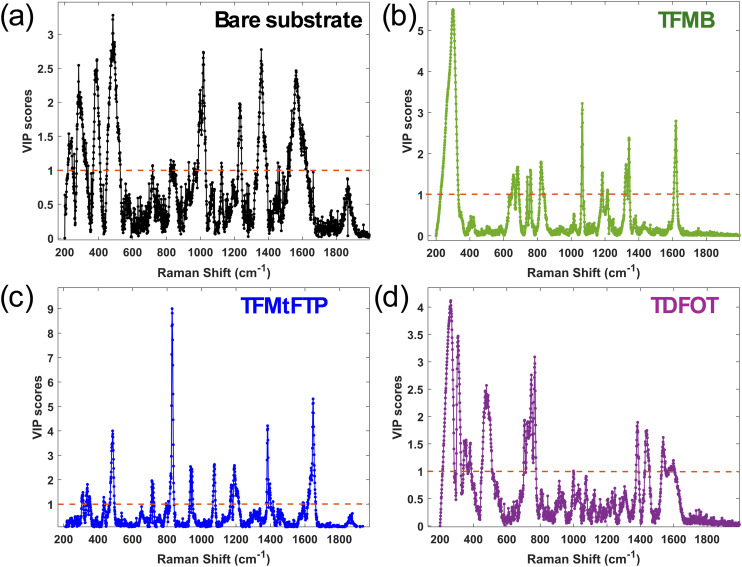
VIP scores of the PLS regression models constructed from PFOA interaction on (a) bare substrate (b) TFMB (c) TFMtFTP and (d) TDFOT modified substrates. Red dotted line shows the significance threshold.

The VIP score analysis reveals that different surface modifications lead to distinct spectral signatures in PFOA detection (Fig. 6). The bare gold substrate shows the highest VIP scores observed in the regions of 1015 cm^−1^ and 1232, 1360, and 1560 cm^−1^ (Fig. 6a). As discussed in Fig. 3a, these spectral regions are likely associated with vibrational modes of gold or methanol.^25,65^ A high VIP score was observed in the lower region at 290 cm^−1^ and 520 cm^−1^, corresponding to Au–S bonding and the Si substrate, as well as at 382 cm^−1^, which is attributed to CF_2_ twisting.^61^ These peaks are also present at 0 nM PFOA, indicating that they are not associated with PFOA vibrational modes. However, the observed alterations in these regions can be attributed to the interaction between PFOA and the substrate or solvent molecules. This interaction leads to subtle changes in the local chemical environment, which are reflected in the SERS spectrum and captured by the VIP scores.

In contrast, the thiol-modified surfaces demonstrate distinct spectral patterns, indicating interactions between the different thiol moieties and PFOA molecules. TDFOT shows significant VIP scores in the 290–477 cm^−1^, 700–770 cm^−1^, 1350–1450 cm^−1^ and 1538 cm^−1^ regions (Fig. 6d). These regions likely represent various C–F vibrations,^66^ and C=C stretching^46^ suggesting strong fluorine–fluorine interactions between TDFOT and PFOA, which aligns with its molecular structure. The TFMB modification shows prominent VIP scores in the 660–826 cm^−1^, 1070 cm^−1^, and 1340 and 1600 cm^−1^ regions (Fig. 6b). These regions may indicate C–S, C–F, C=C, and potential π–π interactions between the TFMB aromatic ring and PFOA, explaining its lower error value. The moderate performance of TFMtFTP is reflected in its VIP score profile, which shows a mix of thiol and fluorine interactions (Fig. 6c). High VIP scores in the 470 cm^−1^, 700–800 cm^−1^, 1070–1190 cm^−1^, 1384 and 1640 cm^−1^ regions might represent C–S stretching, C–F stretching and C=C stretching, respectively, indicating thiol–PFOA interactions.

The superior performance of TDFOT modifications can be attributed to their molecular structures and interactions with PFOA. TDFOT, being a long-chain fluorinated thiol, likely provides a more favorable environment for PFOA molecules, enhancing the SERS effect and improving detection sensitivity. TFMB and TFMtFTP, despite aromatic structure, also demonstrates strong affinity for PFOA, possibly due to PFOA altering π–π interactions between thiols. The moderate improvement seen with TFMtFTP suggests that while it does enhance PFOA detection compared to the bare substrate, its molecular structure may not be as optimal for PFOA interactions as TDFOT.

Analysis of the model interpretability outputs revealed that the spectral regions most heavily weighted by the ML models correspond to fluorinated functional groups in PFOA. The VIP scores showed dominant contributions in the 700–780 cm^−1^ region (CF_2_ deformation), 1070–1190 cm^−1^ region (CF_2_ stretching), and 1340–1390 cm^−1^ region (CF_3_ symmetric stretching). These vibrational bands are well-established PFOA markers based on prior Raman/DFT studies.^24,61^ Their prominence in the ML feature importance analysis indicates that the models rely on C–F and CF_2_/CF_3_ vibrational signatures to distinguish PFAS species and concentrations. This is consistent with the proposed fluorophilic interaction mechanism, whereby selective adsorption and orientation of PFOA on the functionalized SERS surface enhances these characteristic fluorinated-group modes.

### PFOA detection in water

**Fig. 7:**
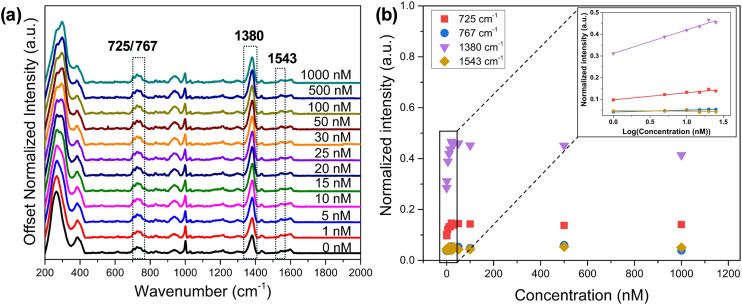
(a) SERS spectra of PFOA in water at concentrations ranging from 0 to 1 μM on the TDFOT-modified SERS substrate. (b) Corresponding dose–response curves for the Raman bands at 725, 767, 1380, and 1543 cm^−1^. The inset in (b) shows the dose–response curve highlighting the lower concentration range.

Experiments were performed to demonstrate that Raman active modes resulting from ligand–PFOA interactions could be used to detect PFOA in water. The ligand TDFOT was selected based on its performance identified through machine learning, specifically the VIP scores from the PLSR model. Fig. 7a presents the normalized SERS spectra of PFOA in water, while Fig. 7b illustrates the corresponding response curves of key Raman bands as a function of concentration. As shown in Fig. 7b, the signal intensity increased with increasing PFOA concentration and exhibited a semi-log linear relationship up to 25 nM. The calibration curves for the Raman bands at 725, 767, 1380, and 1543 cm^−1^ exhibited *R*^2^ values of 0.94, 0.94, 0.98, and 0.10, respectively. Beyond this concentration, the normalized intensity reached a plateau, consistent with surface saturation (see SI). The most prominent responses that align with high VIP scores were observed at 725 cm^−1^ (and to a lesser extent at 767 cm^−1^) which fall within the region 700–780 cm^−1^ for CF_2_ deformation, and at 1380 cm^−1^, which falls within 1340–1390 cm^−1^ for CF_3_ symmetric stretching.

Overall, the ligand–PFOA interactions produce distinct and concentration-dependent Raman signatures that enable the detection of PFOA in deionized water. The strong agreement between experimentally responsive Raman bands and those identified by the PLSR–VIP analysis further validates the machine learning-guided ligand selection strategy. The observed linear response at environmentally relevant concentrations, followed by saturation behavior at higher levels, underscores the robustness of the SERS platform while highlighting its potential applicability for quantitative monitoring of PFOA in water.

## Conclusions

This study demonstrates the potential of combining fluorinated thiol-modified SERS substrates with ML techniques for enhanced detection based on of PFAS–ligand interactions. By leveraging fluorinated thiols as surface modifiers and applying advanced ML models, our approach addresses key gaps in current research related to fluorophilic interactions. This strategy improved detection sensitivity by a factor of up to 2.78 compared to bare substrates, achieving a limit of detection of 3.6 ppb for PFOA in methanol. The high classification accuracy for sample groups demonstrates the power of combining SERS with machine learning for complex environmental sample analysis. The insights obtained from this study offer a valuable framework for understanding fluorophilic interactions at functionalized SERS surfaces, which can inform the rational design of next-generation SERS-based detection platforms with enhanced sensitivity for PFOA. We further validated the proposed machine learning guided SERS approach for PFOA detection directly in water, demonstrating ligand-specific Raman responses arising from ligand–PFOA interactions. The method exhibited a linear and sensitive response at environmentally relevant concentrations, confirming its applicability for aqueous PFAS monitoring. By demonstrating the potential of fluorinated thiol-modified substrates in combination with ML tools, this work lays important groundwork for advancing PFAS detection technologies.

The absence of experimental anti-interference validation and real sample testing is a notable limitation. These aspects are critical for establishing the robustness, and selectivity of the proposed method in realistic, complex sample matrices where co-contaminants may affect sensor performance. Future work should focus on expanding this approach to a broader range of PFAS compounds and exploring its applicability in complex environmental matrices, further validating their suitability for real-world detection scenarios. Through continued refinement and validation, this SERS-based platform has the potential to become a versatile and powerful tool for environmental PFAS monitoring.

## Author contributions

Dr. M. Poonia contributed to the conceptualization, data curation, formal analysis, investigation, methodology, validation, visualization, and writing (original draft). K. Terceiro contributed to the formal analysis, investigation, validation, visualization, and writing (review and editing). Dr. G. Bothun contributed to the conceptualization, funding acquisition, methodology, project administration, supervision, and writing (review and editing).

## Conflicts of interest

There are no conflicts to declare.

## Supplementary Material

EN-013-D5EN00721F-s001

## Data Availability

Data for this article, including raw data for Raman spectra and X-ray photoelectron spectra, are available at DigitalCommons@URI at https://digitalcommons.uri.edu/data/. Supplementary information (SI): the SI contains additional figures and tables that complement the main text. Fig. S1–S5 include SERS intensity plots, ROC curves, regression parity plots, and PLS-DA score and cross-validation plots for bare and thiol-functionalized substrates. Tables S1–S2 provide Raman peak assignments for fluorinated thiols and PFOA, and a summary of reported PFOA/PFOS detection limits from the literature. See DOI: https://doi.org/10.1039/d5en00721f.

## References

[cit1] Lohmann R., Cousins I. T., Dewitt J. C., Glüge J., Goldenman G., Herzke D., Lindstrom A. B., Miller M. F., Ng C. A., Patton S. (2020). *et al.*, Are Fluoropolymers Really of Low Concern for Human and Environmental Health and Separate from Other PFAS?. Environ. Sci. Technol..

[cit2] Menger R. F., Funk E., Henry C. S., Borch T. (2021). Sensors for detecting per- and polyfluoroalkyl substances (PFAS): A critical review of development challenges, current sensors, and commercialization obstacles. Chem. Eng. J..

[cit3] Medina H., Farmer C. (2024). Current Challenges in Monitoring Low Contaminant Levels of Per- and Polyfluoroalkyl Substances in Water Matrices in the Field. Toxics.

[cit4] McDonnell C., Albarghouthi F. M., Selhorst R., Kelley-Loughnane N., Franklin A. D., Rao R. (2023). Aerosol Jet Printed Surface-Enhanced Raman Substrates: Application for High-Sensitivity Detection of Perfluoroalkyl Substances. ACS Omega.

[cit5] Wang Y., Darling S. B., Chen J. (2021). Selectivity of Per- and Polyfluoroalkyl Substance Sensors and Sorbents in Water. ACS Appl. Mater. Interfaces.

[cit6] Srivastava S., Wang W., Zhou W., Jin M., Vikesland P. J. (2024). Machine Learning-Assisted Surface-Enhanced Raman Spectroscopy Detection for Environmental Applications: A Review. Environ. Sci. Technol..

[cit7] Rothstein J. C., Cui J., Yang Y., Chen X., Zhao Y. (2024). Ultra-sensitive detection of PFASs using surface enhanced Raman scattering and machine learning: a promising approach for environmental analysis. Sens. Diagn..

[cit8] Poonia M., Morder C. J., Schorr H. C., Schultz Z. D. (2024). Raman and Surface-Enhanced Raman Scattering Detection in Flowing Solutions for Complex Mixture Analysis. Annu. Rev. Anal. Chem..

[cit9] Kuster T., Bothun G. D. (2021). In situ SERS detection of dissolved nitrate on hydrated gold substrates. Nanoscale Adv..

[cit10] Poonia M., Kurtz K., Green-Gavrielidis L., Oyanedel-Craver V., Bothun G. D. (2023). Electric Potential Induced Prevention and Removal of an Algal Biofoulant from Planar SERS Substrates. Environ. Sci. Technol..

[cit11] Poonia M., Küster T., Bothun G. D. (2022). Organic Anion Detection with Functionalized SERS Substrates via Coupled Electrokinetic Preconcentration, Analyte Capture, and Charge Transfer. ACS Appl. Mater. Interfaces.

[cit12] Langer J., Jimenez de Aberasturi D., Aizpurua J., Alvarez-Puebla R. A., Auguie B., Baumberg J. J., Bazan G. C., Bell S. E. J., Boisen A., Brolo A. G. (2020). *et al.*, Present and Future of Surface-Enhanced Raman Scattering. ACS Nano.

[cit13] Fang C., Megharaj M., Naidu R. (2016). Surface-enhanced Raman scattering (SERS) detection of fluorosurfactants in firefighting foams. RSC Adv..

[cit14] Park H., Park J., Kim W., Kim W., Park J. (2023). Ultra-sensitive SERS detection of perfluorooctanoic acid based on self-assembled p-phenylenediamine nanoparticle complex. J. Hazard. Mater..

[cit15] Feng Y., Dai J., Wang C., Zhou H., Li J., Ni G., Zhang M., Huang Y. (2023). Ag Nanoparticle/Au@Ag Nanorod Sandwich Structures for SERS-Based Detection of Perfluoroalkyl Substances. ACS Appl. Nano Mater..

[cit16] Lada Z. G., Mathioudakis G. N., Soto Beobide A., Andrikopoulos K. S., Voyiatzis G. A. (2024). Generic method for the detection of short & long chain PFAS extended to the lowest concentration levels of SERS capability. Chemosphere.

[cit17] Yang Z., Zhu Y., Tan X., Gunjal S. J. J., Dewapriya P., Wang Y., Xin R., Fu C., Liu K., Macintosh K. (2024). *et al.*, Fluoropolymer sorbent for efficient and selective capturing of per- and polyfluorinated compounds. Nat. Commun..

[cit18] Moro G., Dongmo Foumthuim C. J., Spinaci M., Martini E., Cimino D., Balliana E., Lieberzeit P., Romano F., Giacometti A., Campos R. (2022). *et al.*, How perfluoroalkyl substances modify fluorinated self-assembled monolayer architectures: An electrochemical and computational study. Anal. Chim. Acta.

[cit19] He Y., Cheng X., Gunjal S. J., Zhang C. (2024). Advancing PFAS Sorbent Design: Mechanisms, Challenges, and Perspectives. ACS Mater. Au.

[cit20] Roman Santiago A., Yin S., Elbert J., Lee J., Shukla D., Su X. (2023). Imparting Selective Fluorophilic Interactions in Redox Copolymers for the Electrochemically Mediated Capture of Short-Chain Perfluoroalkyl Substances. J. Am. Chem. Soc..

[cit21] Ding Y., Sun Y., Liu C., Jiang Q. Y., Chen F., Cao Y. (2023). SERS-Based Biosensors Combined with Machine Learning for Medical Application. ChemistryOpen.

[cit22] Jiang T., Gradus J. L., Rosellini A. J. (2020). Supervised Machine Learning: A Brief Primer. Behav. Ther..

[cit23] Kwon H., Ali Z. A., Wong B. M. (2023). Harnessing Semi-Supervised Machine Learning to Automatically Predict Bioactivities of Per- and Polyfluoroalkyl Substances (PFASs). Environ. Sci. Technol. Lett..

[cit24] Cho S., Remucal C. K., Wei H. (2025). Common and Distinctive Raman Spectral Features for the Identification and Differentiation of Per- and Polyfluoroalkyl Substances. ACS ES&T Water.

[cit25] Schmidt M. S., Hubner J., Boisen A. (2012). Large area fabrication of leaning silicon nanopillars for surface enhanced Raman spectroscopy. Adv. Mater..

[cit26] Muñoz P., Noordam C. T. N., Egberink R. J. M., Huskens J., Garcia-Blanco S. M. (2019). Comparative study of multiple thiol-based self-assembled monolayer coatings for the SERS detection of nitrite, nitrate, and perchlorate anions in water. Appl. Opt..

[cit27] Chevalier R. B., Dwyer J. R. (2021). Optimizing noncontact oxygen-plasma treatment to improve the performance of a top-down nanofabricated surface enhanced Raman spectroscopy substrate with structurally responsive, high-aspect-ratio nanopillar array. J. Raman Spectrosc..

[cit28] Hinterwirth H., Kappel S., Waitz T., Prohaska T., Lindner W., Lämmerhofer M. (2013). Quantifying Thiol Ligand Density of Self-Assembled Monolayers on Gold Nanoparticles by Inductively Coupled Plasma–Mass Spectrometry. ACS Nano.

[cit29] Love J. C., Estroff L. A., Kriebel J. K., Nuzzo R. G., Whitesides G. M. (2005). Self-Assembled Monolayers of Thiolates on Metals as a Form of Nanotechnology. Chem. Rev..

[cit30] Küster T., Bothun G. D. (2025). Self-Assembled Cysteamine Reporter Ligands for SERS Nitrate Detection in Continuous Flow. Langmuir.

[cit31] Brandt N. N., Brovko O. O., Chikishev A. Y., Paraschuk O. D. (2006). Optimization of the Rolling-Circle Filter for Raman Background Subtraction. Appl. Spectrosc..

[cit32] Lee L. C., Liong C.-Y., Jemain A. A. (2018). Partial least squares-discriminant analysis (PLS-DA) for classification of high-dimensional
(HD) data: a review of contemporary practice strategies and knowledge gaps. Analyst.

[cit33] Caporali S., Muniz-Miranda F., Pedone A., Muniz-Miranda M. (2019). SERS, XPS and DFT Study of Xanthine Adsorbed on Citrate-Stabilized Gold Nanoparticles. Sensors.

[cit34] Sarma D., Nath K. K., Biswas S., Chetia I., Badwaik L. S., Ahmed G. A., Nath P. (2023). SERS determination and multivariate classification of antibiotics in chicken meat using gold nanoparticle-decorated electrospun PVA nanofibers. Microchim. Acta.

[cit35] Ranganathan K., Morais A., Nongwe I., Longo C., Nogueira A. F., Coville N. J. (2016). Study of photoelectrochemical water splitting using composite films based on TiO2 nanoparticles and nitrogen or boron doped hollow carbon spheres as photoanodes. J. Mol. Catal. A: Chem..

[cit36] Gengenbach T. R., Major G. H., Linford M. R., Easton C. D. (2021). Practical guides for x-ray photoelectron spectroscopy (XPS): Interpreting the carbon 1s spectrum. J. Vac. Sci. Technol., A.

[cit37] Fujimoto A., Yamada Y., Koinuma M., Sato S. (2016). Origins of sp(3)C peaks in C1s X-ray Photoelectron Spectra of Carbon Materials. Anal. Chem..

[cit38] Guselnikova O., Svorcik V., Lyutakov O., Chehimi M. M., Postnikov P. S. (2019). Preparation of Selective and Reproducible SERS Sensors of Hg2+ Ions via a Sunlight-Induced Thiol–Yne Reaction on Gold Gratings. Sensors.

[cit39] Sosunov A. V., Ziolkowska D. A., Ponomarev R. S., Henner V. K., Karki B., Smith N., Sumanasekera G., Jasinski J. B. (2019). CFx primary batteries based on fluorinated carbon nanocages. New J. Chem..

[cit40] Lee J.-W., Jeong S.-P., You N.-H., Moon S.-Y. (2021). Tunable Synthesis of Predominant Semi-Ionic and Covalent Fluorine Bonding States on a Graphene Surface. Nanomaterials.

[cit41] Ruzicka J.-Y., Bakar F. A., Thomsen L., Cowie B. C., McNicoll C., Kemmitt T., Brand H. E. A., Ingham B., Andersson G. G., Golovko V. B. (2014). XPS and NEXAFS study of fluorine modified TiO2 nano-ovoids reveals dependence of Ti3+ surface population on the modifying agent. RSC Adv..

[cit42] Varnholt B., Oulevey P., Luber S., Kumara C., Dass A., Bürgi T. (2014). Structural Information on the Au–S Interface of Thiolate-Protected Gold Clusters: A Raman Spectroscopy Study. J. Phys. Chem. C.

[cit43] Bandyopadhyay S., Chattopadhyay S., Dey A. (2015). The protonation state of thiols in self-assembled monolayers on roughened Ag/Au surfaces and nanoparticles. Phys. Chem. Chem. Phys..

[cit44] Li C., Fang X., Li H., Zhang X. (2024). Direct and Rapid Sensing of Per- and Polyfluoroalkyl Substances Using SERS-Active Optical Fibers. ACS Appl. Opt. Mater..

[cit45] Even M. A., Lee S. H., Wang J., Chen Z. (2006). Detection and spectral analysis of trifluoromethyl groups at a surface by sum frequency generation vibrational spectroscopy. J. Phys. Chem. B.

[cit46] Karnan M., Balachandran V., Murugan M., Murali M. K., Nataraj A. (2013). Vibrational (FT-IR and FT-Raman) spectra, NBO, HOMO-LUMO, Molecular electrostatic potential surface and computational analysis of 4-(trifluoromethyl)benzylbromide. Spectrochim. Acta, Part A.

[cit47] Boehmke Amoruso A., Boto R. A., Elliot E., de Nijs B., Esteban R., Foldes T., Aguilar-Galindo F., Rosta E., Aizpurua J., Baumberg J. J. (2024). Uncovering low-frequency vibrations in surface-enhanced Raman of organic molecules. Nat. Commun..

[cit48] Madzharova F., Heiner Z., Kneipp J. (2020). Surface-Enhanced Hyper Raman Spectra of Aromatic Thiols on Gold and Silver Nanoparticles. J. Phys. Chem. C.

[cit49] Hong Y., Wang R., Jiang Z., Cong Z., Song H. (2020). Rapid SERS Detection of Thiol-Containing Natural Products in Culturing Complex. Int. J. Anal. Chem..

[cit50] Sibug-Torres S. M., Grys D.-B., Kang G., Niihori M., Wyatt E., Spiesshofer N., Ruane A., de Nijs B., Baumberg J. J. (2024). In situ electrochemical regeneration of nanogap hotspots for continuously reusable ultrathin SERS sensors. Nat. Commun..

[cit51] Goldmann C., Lazzari R., Paquez X., Boissière C., Ribot F., Sanchez C., Chanéac C., Portehault D. (2015). Charge Transfer at Hybrid Interfaces: Plasmonics of Aromatic Thiol-Capped Gold Nanoparticles. ACS Nano.

[cit52] Rittikulsittichai S., Park C. S., Jamison A. C., Rodriguez D., Zenasni O., Lee T. R. (2017). Bidentate Aromatic Thiols on Gold: New Insight Regarding the Influence of Branching on the Structure, Packing, Wetting, and Stability of Self-Assembled Monolayers on Gold Surfaces. Langmuir.

[cit53] Gnyba M., Wierzba P., Smulko J., Kwiatkowski A. (2011). Portable Raman spectrometer - design rules and applications. Bull. Pol. Acad. Sci.: Tech. Sci..

[cit54] Cardellini J., Dallari C., De Santis I., Riccio L., Ceni C., Morrone A., Calamai M., Pavone F. S., Credi C., Montis C. (2024). *et al.*, Hybrid lipid-AuNP clusters as highly efficient SERS substrates for biomedical applications. Nat. Commun..

[cit55] Pérez-Jiménez A. I., Lyu D., Lu Z., Liu G., Ren B. (2020). Surface-enhanced Raman spectroscopy: benefits, trade-offs and future developments. Chem. Sci..

[cit56] Fang C., Sobhani Z., Megharaj M., Naidu R. (2018). Electrochemical Proof of Fluorophilic Interaction among Fluoro-Carbon Chains. Electroanalysis.

[cit57] Fu K., Huang J., Luo F., Fang Z., Yu D., Zhang X., Wang D., Xing M., Luo J. (2024). Understanding the Selective Removal of Perfluoroalkyl and Polyfluoroalkyl Substances via Fluorine–Fluorine Interactions: A Critical Review. Environ. Sci. Technol..

[cit58] Manayil Parambil A., Priyadarshini E., Paul S., Bakandritsos A., Sharma V. K., Zbořil R. (2025). Emerging nanomaterials for the detection of per- and poly-fluorinated substances. J. Mater. Chem. A.

[cit59] Lasalvia M., Capozzi V., Perna G. (2022). A Comparison of PCA-LDA and PLS-DA Techniques for Classification of Vibrational Spectra. Appl. Sci..

[cit60] Mehmood N., Akram M. W., Majeed M. I., Nawaz H., Aslam M. A., Naman A., Wasim M., Ghaffar U., Kamran A., Nadeem S. (2024). *et al.*, Surface-enhanced Raman spectroscopy for the characterization of bacterial pellets of Staphylococcus aureus infected by bacteriophage. RSC Adv..

[cit61] Kumar A., Rothstein J. C., Chen Y., Zhang H., Zhao Y. (2025). Experimental Raman spectra analysis of selected PFAS compounds:
Comparison with DFT predictions. J. Hazard. Mater..

[cit62] Ostra M., Ubide C., Vidal M., Zuriarrain J. (2008). Detection limit estimator for multivariate calibration by an extension of the IUPAC recommendations for univariate methods. Analyst.

[cit63] Allegrini F., Olivieri A. C. (2014). IUPAC-Consistent Approach to the Limit of Detection in Partial Least-Squares Calibration. Anal. Chem..

[cit64] Despagne F., Massart D.-L., de Noord O. E. (1997). Optimization of Partial-Least-Squares Calibration Models by Simulation of Instrumental Perturbations. Anal. Chem..

[cit65] Vašková H., Tomeček M. (2018). Rapid spectroscopic measurement of methanol in water-ethanol-methanol mixtures. MATEC Web Conf..

[cit66] Bhavya M. B., Jena S. R., Yadav S., Altaee A., Saxena M., Samal A. K. (2023). Detection of PFAS via surface-enhanced Raman scattering: Challenges and future perspectives. Sustainable Chem. Environ..

