# Supplementary material for: Machine learning-enhanced identification of fluorophilic interactions for improved SERS detection of PFOA
Source: Environ Sci Nano. 2026 Feb 20;13(4):2003–17. doi: 10.1039/d5en00721f (PMC12980129; doi:10.1039/d5en00721f)
Supplement: EN-013-D5EN00721F-s001 [file EN-013-D5EN00721F-s001.pdf]

Supplementary Information (SI) for Environmental Science: Nano.
This journal is © The Royal Society of Chemistry 2026

Machine Learning-Enhanced Identification of Fluorophilic Interactions for Improved SERS Detection of PFOA

Monika Poonia[a,*], Kathryn Terceiro[b], Geoffrey D. Bothun[b,*]

[a]Department of Civil and Environmental Engineering, Wayne State University, Detroit, MI 48202, United States

[b]Department of Chemical, Biomolecular, and Materials Engineering, University of Rhode Island, Kingston, RI 02881, United States

*Co-corresponding authors: Monika Poonia, hx1590@wayne.edu; Geoffrey D. Bothun, gbothun@uri.edu

List of supplementary figures

Figure S1. PFOA concentration vs SERS intensity on (a) bare substrate (b) TFMB (c) TFMtFTP and (d) TDFOT modified substrates. Error bar represents the spectral variance observed between replicate measurements.

Figure S2. Receiver operating characteristics (ROC) curve demonstrating classification capability of PLS-DA classification model at (a) bare substrate (b) TFMB (c) TFMtFTP and (d) TDFOT modified substrates.

Figure S3: Cross-validation parity plots showing the predicted concentration versus the measured concentration using SVM Regression in PFOA spectra on (a) bare substrate (b) TFMB (c) TFMtFTP and (d) TDFOT modified substrates. The fit line (red) and 1:1 line (green) represents the overall trend of the model's predictions.

Figure S4: PLS-DA score plots for the four substrate conditions (a) Bare substrate, (b) TFMB, (c) TFMtFTP, and (d) TDFOT modified substrates comparing spectra for before (only methanol, blue diamonds) and after (PFOA, red squares) exposure.

Figure S5: Cross-validated Y-prediction plots from PLS-DA models for all four substrate conditions (a) Bare substrate, (b) TFMB, (c) TFMtFTP, and (d) TDFOT modified substrates comparing spectra for before (only methanol, blue diamonds) and after (PFOA, red squares) exposure.

List of supplimentary tables

Table S1. An assignment of the major Raman peaks in fluorinated thiols and PFOA

Table S2. Summary of available literature on the detection of PFOA/PFOS via SERS and mass spectroscopy along with respective LODs.

Table S1. An assignment of the major Raman peaks in gold-coated SERS substrate, fluorinated thiols and PFOA.

Wavenumber (cm^{-1})	Peak assignment	Reference
290	C-C, CF_2	[1]
311	Au-S bending	[2, 3]
337	Au-S bending	[2]
382	CF_2 twisting	[1, 4]
412	C-S Stretching	[5]
477	C-S_G	[2]
520	Si surface	[6, 7]
632/649	C-S_G	[5, 8]
673	C-S_T	[5, 8]
720	C-S_T, C-C, CF_3	[4, 5, 9]
748	C-C, CF_3	[9]
767	CF3 Stretching	[9, 10]
828	CF3 Stretching	[11]
1000	C-C aromatic	[12]
1074	C-F Stretching	[9, 10]
1120	C-F Stretching, CF_2	[10]
1136/1147	C-F Stretching, C-C	[4, 9, 10, 13]
1175/1185	C-F Stretching	[9, 10]
1222	C-F Stretching	[9]
1340	C-F Stretching	[9]
1360	C-F Stretching, CF_3	[9]
1390	C-F Stretching, COO, CF_3	[9, 10, 13]
1420	C-F Stretching	[9]
1538	C=C Stretching	[12]
1611	C=C Stretching	[12, 14]
1643	C=C Stretching	[12]

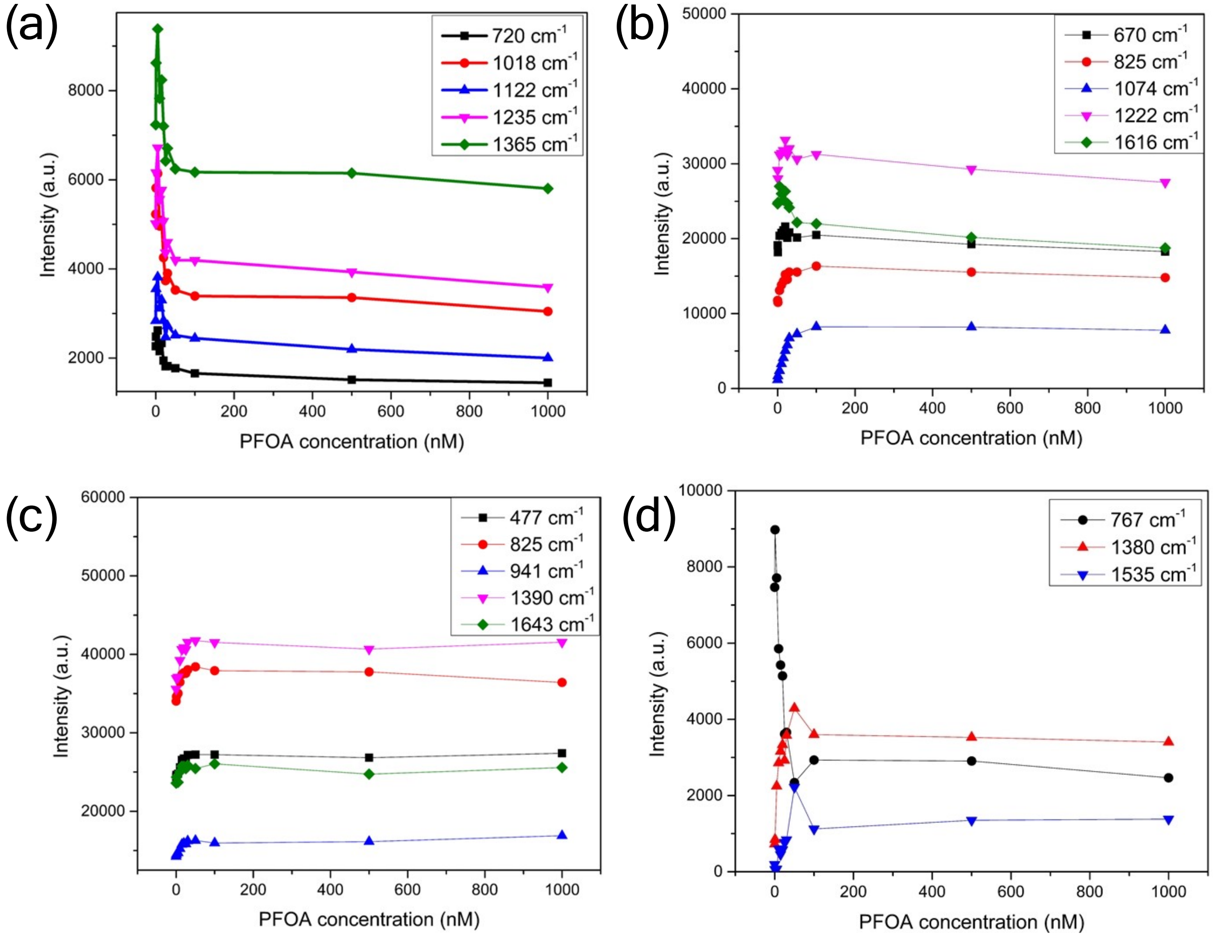

Figure S1. PFOA concentration vs SERS intensity on (a) bare substrate (b) TFMB (c) TFMtFTP and (d) TDFOT modified substrates. Error bar represents the spectral variance observed between replicate measurements.

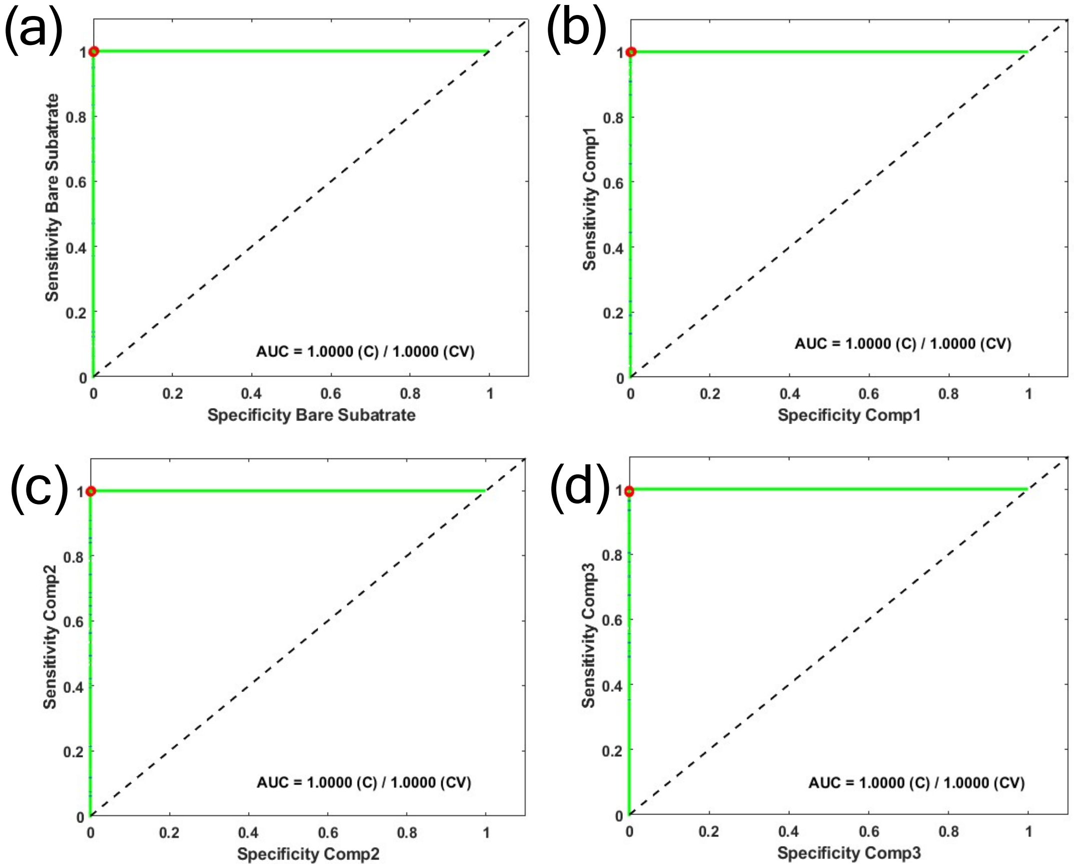

Figure S2. Receiver operating characteristics (ROC) curve demonstrating classification capability of PLS-DA classification model at (a) bare substrate (b) TFMB (c) TFMtFTP and (d) TDFOT modified substrates.

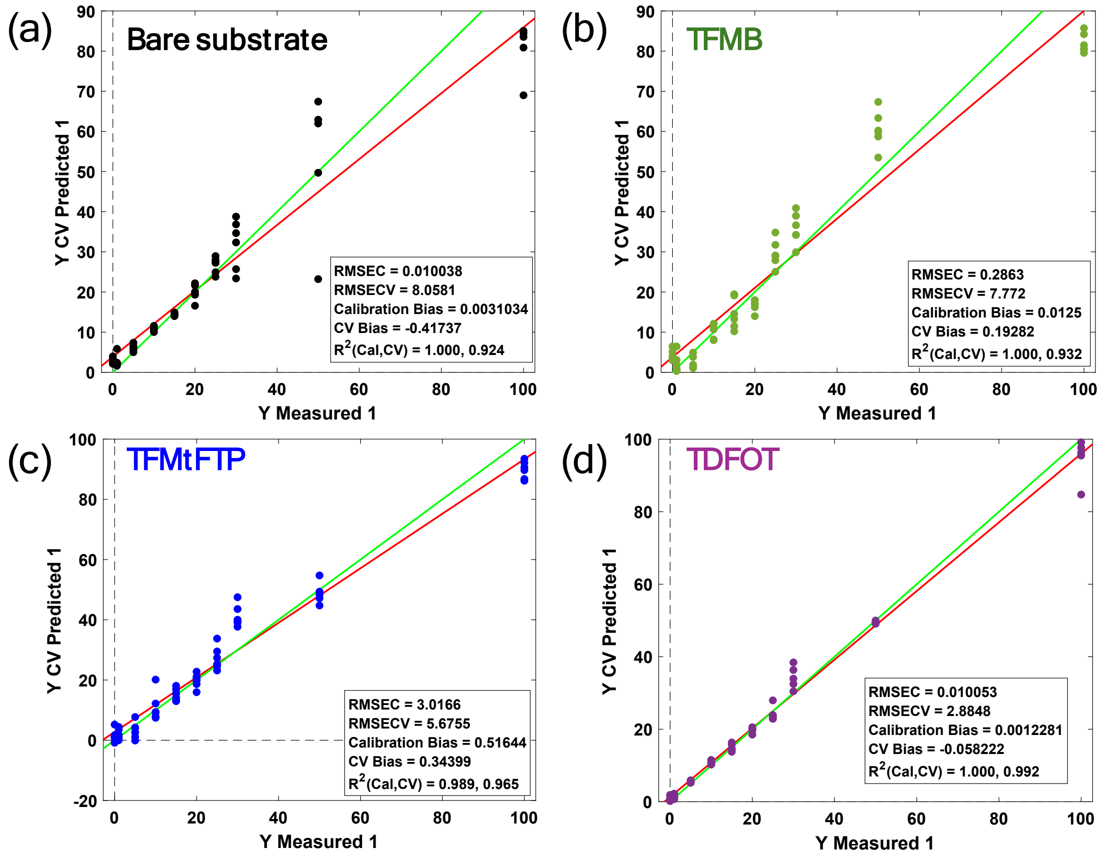

Figure S3: Cross-validation parity plots showing the predicted concentration versus the measured concentration using SVM Regression in PFOA spectra on (a) bare substrate (b) TFMB (c) TFMtFTP and (d) TDFOT modified substrates. The fit line (red) and 1:1 line (green) represents the overall trend of the model's predictions.

Table S2. Summary of available literature on the detection of PFOA/PFOS via SERS and mass spectroscopy along with respective LODs.

**Detection methods and substrates**	**LOD**	**Reference**
Ag nanograss	1.28 pM	[15]
Ag NPs + Graphene oxide	50 ppb	[16]
Ag NPs + β-cyclodextrin	40 ng/L	[4]
AgNPs/Au@AgNRs sandwich	100 ppb	[17]
AgNPs + Graphene	0.4 ppt	[18]
AgNR + alkanethiol	1 ppt	[9]
Ag nanocolloidal	5 ppt	[19]
PFAS in wastewater by liquid chromatography-mass spectrometry (LC-MS)	0.1 ng mL^{-1}	[20]
PFAS in soil sample using paper spray (PS)-based MS	1.2–4.5 ppt	[19]
PFOS in water using MS	5.6 ppm	[21]
PFASs in drinking, sea and surface water using LC-MS	0.5–17 ng L^{-1}	[22]
Au substrate + Fluorinated thiol	3.6 ppb	This study

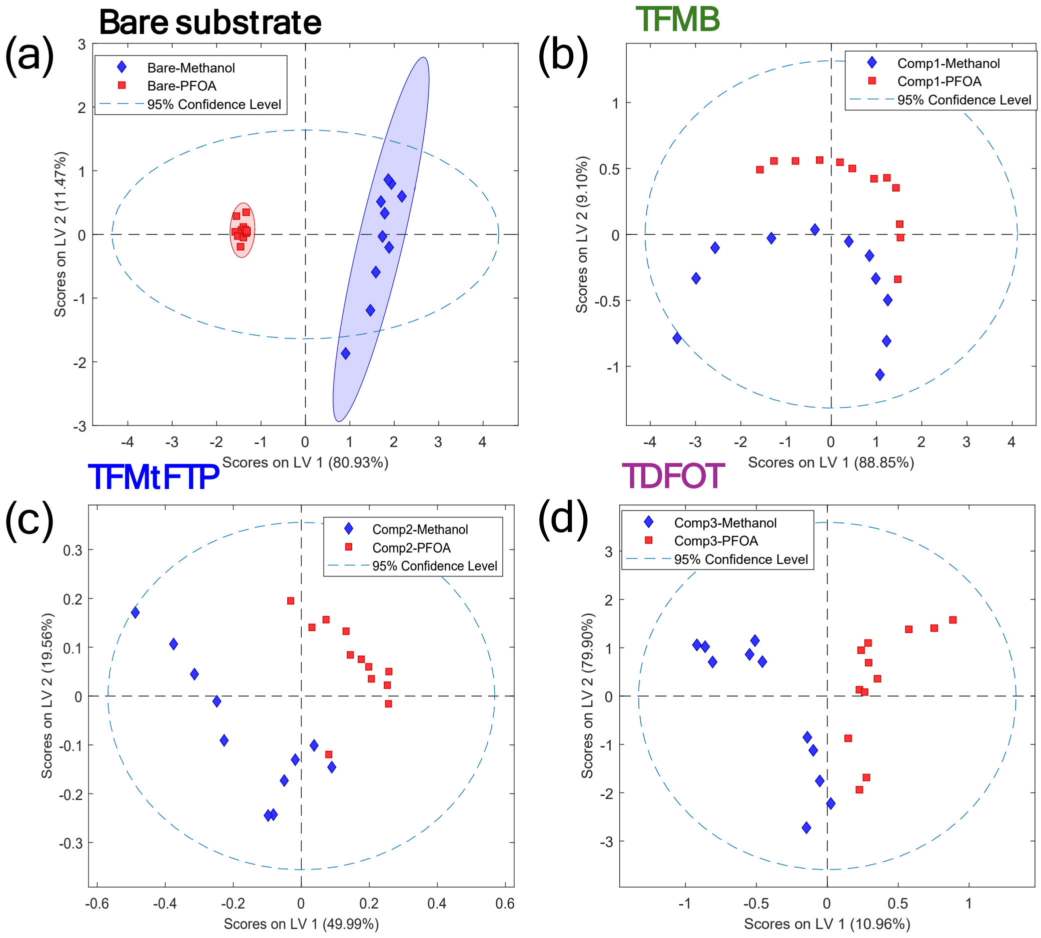

Figure S4. PLS-DA score plots for the four substrate conditions (a) Bare substrate, (b) TFMB, (c) TFMtFTP, and (d) TDFOT modified substrates comparing spectra for before (only methanol, blue diamonds) and after (PFOA, red squares) exposure.

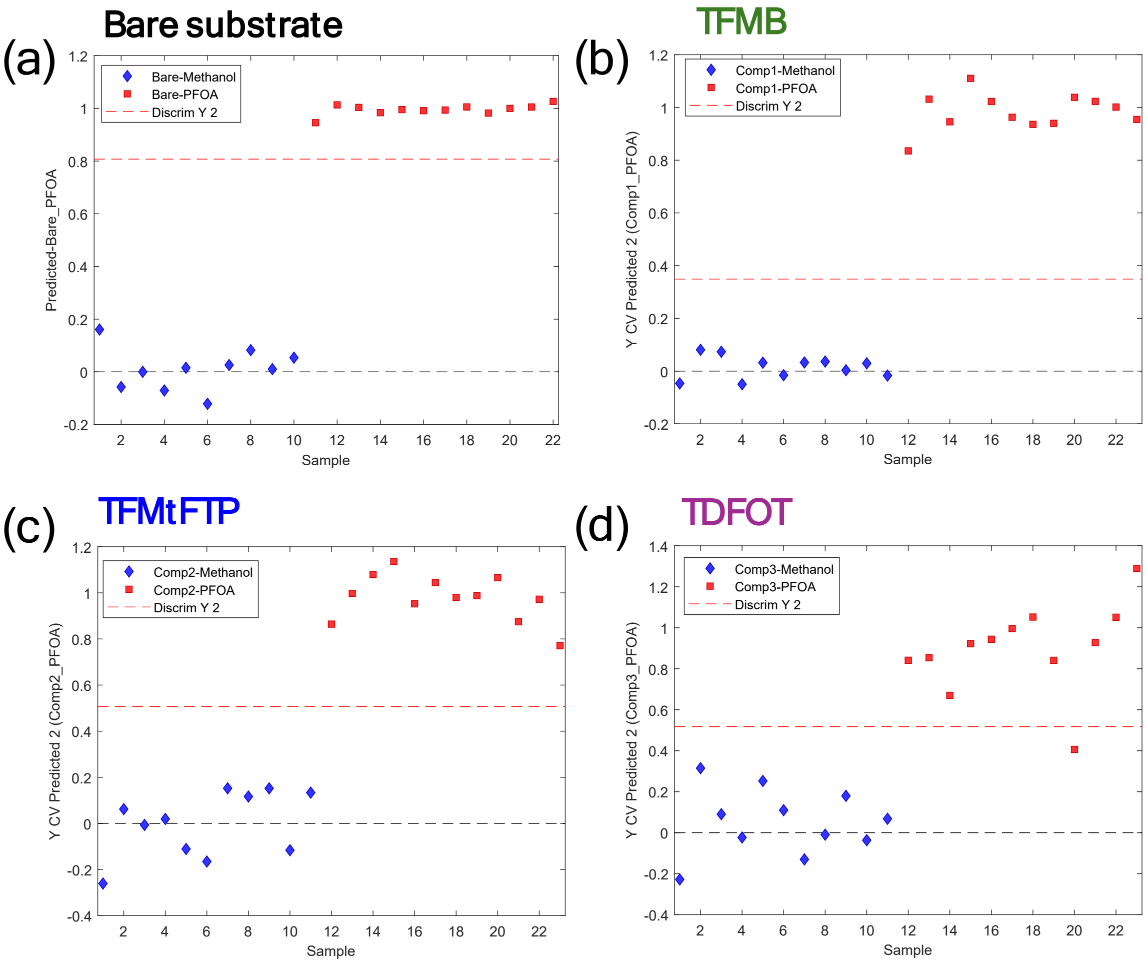

Figure S5. Cross-validated Y-prediction plots from PLS-DA models for all four substrate conditions (a) Bare substrate, (b) TFMB, (c) TFMtFTP, and (d) TDFOT modified substrates comparing spectra for before (only methanol, blue diamonds) and after (PFOA, red squares) exposure.

References

1. Cho, S., C.K. Remucal, and H. Wei, *Common and Distinctive Raman Spectral Features for the Identification and Differentiation of Per- and Polyfluoroalkyl Substances.* ACS ES&T Water, 2025. **5**(1): p. 300–309.
2. Varnholt, B., et al., *Structural Information on the Au–S Interface of Thiolate-Protected Gold Clusters: A Raman Spectroscopy Study.* The Journal of Physical Chemistry C, 2014. **118**(18): p. 9604–9611.
3. Dolamic, I., B. Varnholt, and T. Bürgi, *Far-infrared spectra of well-defined thiolate-protected gold clusters.* Physical Chemistry Chemical Physics, 2013. **15**(45): p. 19561–19565.
4. Li, C., et al., *Direct and Rapid Sensing of Per- and Polyfluoroalkyl Substances Using SERS-Active Optical Fibers.* ACS Applied Optical Materials, 2024. **2**(4): p. 610–616.

5. Bandyopadhyay, S., S. Chattopadhyay, and A. Dey, *The protonation state of thiols in self-assembled monolayers on roughened Ag/Au surfaces and nanoparticles.* Physical Chemistry Chemical Physics, 2015. **17**(38): p. 24866–24873.
6. Kadlečíková, M., et al., *Raman spectroscopy of silicon with nanostructured surface.* Optik, 2022. **257**: p. 168869.
7. Poonia, M., et al., *Electric Potential Induced Prevention and Removal of an Algal Biofoulant from Planar SERS Substrates.* Environ Sci Technol, 2023. **57**(31): p. 11666–11674.
8. Hong, Y., et al., *Rapid SERS Detection of Thiol-Containing Natural Products in Culturing Complex.* Int J Anal Chem, 2020. **2020**: p. 9271236.
9. Rothstein, J.C., et al., *Ultra-sensitive detection of PFASs using surface enhanced Raman scattering and machine learning: a promising approach for environmental analysis.* Sensors & Diagnostics, 2024. **3**(8): p. 1272–1284.
10. M.B, B., et al., *Detection of PFAS via surface-enhanced Raman scattering: Challenges and future perspectives.* Sustainable Chemistry for the Environment, 2023. **3**: p. 100031.
11. Even, M.A., et al., *Detection and spectral analysis of trifluoromethyl groups at a surface by sum frequency generation vibrational spectroscopy.* J Phys Chem B, 2006. **110**(51): p. 26089–97.
12. Karnan, M., et al., *Vibrational (FT-IR and FT-Raman) spectra, NBO, HOMO–LUMO, Molecular electrostatic potential surface and computational analysis of 4-(trifluoromethyl)benzylbromide.* Spectrochimica Acta Part A: Molecular and Biomolecular Spectroscopy, 2013. **116**: p. 84–95.
13. Kumar, A., et al., *Experimental Raman spectra analysis of selected PFAS compounds: Comparison with DFT predictions.* Journal of Hazardous Materials, 2025. **494**: p. 138704.
14. Madzharova, F., Z. Heiner, and J. Kneipp, *Surface-Enhanced Hyper Raman Spectra of Aromatic Thiols on Gold and Silver Nanoparticles.* J Phys Chem C Nanomater Interfaces, 2020. **124**(11): p. 6233–6241.
15. Park, H., et al., *Ultra-sensitive SERS detection of perfluorooctanoic acid based on self-assembled p-phenylenediamine nanoparticle complex.* J Hazard Mater, 2023. **453**: p. 131384.
16. Fang, C., M. Megharaj, and R. Naidu, *Surface-enhanced Raman scattering (SERS) detection of fluorosurfactants in firefighting foams.* RSC Advances, 2016. **6**(14): p. 11140–11145.
17. Feng, Y., et al., *Ag Nanoparticle/Au@Ag Nanorod Sandwich Structures for SERS-Based Detection of Perfluoroalkyl Substances.* ACS Applied Nano Materials, 2023. **6**(15): p. 13974–13983.
18. McDonnell, C., et al., *Aerosol Jet Printed Surface-Enhanced Raman Substrates: Application for High-Sensitivity Detection of Perfluoroalkyl Substances.* ACS Omega, 2023. **8**(1): p. 1597–1605.
19. Lada, Z.G., et al., *Generic method for the detection of short & long chain PFAS extended to the lowest concentration levels of SERS capability.* Chemosphere, 2024. **363**: p. 142916.
20. Tang, C., et al., *Comprehensive characterization of per- and polyfluoroalkyl substances in wastewater by liquid chromatography-mass spectrometry and screening algorithms.* npj Clean Water, 2023. **6**(1): p. 6.

21. Yu, X.-Y., et al., *Molecular detection of per- and polyfluoroalkyl substances in water using time-of-flight secondary ion mass spectrometry.* Frontiers in Chemistry, 2023. **Volume 11 - 2023**.
22. Huerta, B., B. McHugh, and F. Regan, *Development and application of an LC-MS method to the determination of poly- and perfluoroalkyl substances (PFASs) in drinking, sea and surface water samples.* Analytical Methods, 2022. **14**(21): p. 2090–2099.
